# Significance of Cancer Stem Cells in Anti-Cancer Therapies

**Published:** 2016-12-31

**Authors:** Mónica Botelho, Helena Alves

**Affiliations:** 1 INSA, National Institute of Health Dr. Ricardo Jorge, Department of Health Promotion and Chronic Diseases, Rua Alexandre Herculano, Porto, Portugal; 2 I3S, Instituto de Investigação e Inovação da Universidade do Porto, Portugal; 3 Fundação Professor Ernesto Morais, Porto, Portugal

**Keywords:** Stem cells, Cancer, Stem cell markers, Anti-cancer therapies

## Abstract

Stem cells are the focus of cutting edge research interest because of their competence both to self-renew and proliferate, and to differentiate into a variety of tissues, offering enticing prospects of growing replacement organs in vitro, among other possible therapeutic implications.

It is conceivable that cancer stem cells share a number of biological hallmarks that are different from their normal-tissue counterparts and that these might be taken advantage of for therapeutic benefits.

In this review we discuss the significance of cancer stem cells in diagnosis and prognosis of cancer as well as in the development of new strategies for anti-cancer drug design.

## Introduction

A normal stem cell is defined by its dual properties of self-renewal and multilineage differentiation potential, and continuously repopulates the mature cells of the organ system that it serves. Signaling pathways dictate that a stem cell undergoes symmetric division to produce two daughter cells that are either both stem cells or both progenitors, or symmetrically one stem cell and one progenitor cell. Through this process, the division of a stem cell results in the formation of two daughter cells – one of which is another stem cell, and the other of which is a committed progenitor that is capable of further differentiation and proliferation but lacks the ability to self-renew [1,2]. Stem cells have many properties that separate them from mature, differentiated cells. In addition to their ability to self-renew and differentiate, they are quiescent, dividing infrequently [3].

In the majority of tissues, stem cells are less than 10 % of total cells. As a result, stem cells must be identified prospectively and isolated carefully in order to study their use. Although it seems reasonable to propose that each tissue arises from tissue-specific stem cell, the rigorous identification and isolation of these somatic stem cells has been accomplished only in a few instances. For example, haematopoietic stem cells (HSCs) have been isolated from mice and humans, and have been shown to be responsible for the generation and regeneration of the blood-forming and immune systems [1,4,5]. Here we review the significance of cancer stem cells in diagnosis and prognosis of cancer as well as in the development of new strategies for anti-cancer drug design.

## Discussion

### Cancer stem cells

This “cancer stem cell hypothesis” represents a remake interpretation of the proposal made by pathologists such as Rudolph Virchow and Julius Cohnheim 150 years ago that “cancer results from the activation of dormant embryonic-tissue remnants” [6,7]. This “embryonal-rest hypothesis” of cancer was based on the histological similarities between the developing fetus and cancer, and the observation that both tissues have an enormous capacity for both proliferation and differentiation, although this differentiation is aberrant in the case of tumors [2].

At the very beginning of life, stem cells can develop into all the different tissues of the body; in contrast, cancer cells often end life. Albeit these unmistakable differences, researchers have suspected that similar mechanisms might be operative in both cancer and stem cells. For instance, both can multiply infinitly. A cancer stem cell would function in a similar way to sustain the growth and spread of tumors while repopulating the distinct cell types represented within the tumor. However, a cancer stem cell would not be subject to the same autologous and heterologous signaling as normal stem cells. The HSC was the first adult somatic stem cell to be described. The existence of cancer stem cells was also first described in the haematopoietic system. Seemingly tumors are composed of a multilineage population of cells, within which resides a small population of cancer stem cells that are solely responsible for the growth and sustenance potential of the whole tumor [2].

There is emerging evidence that stem cell biology could provide new perceptions into cancer biology. In particular, three aspects of the relationship between stem cells and tumor cells are of most importance: first, the similarities in the mechanisms that regulate self-renewal of normal stem cells and cancer cells; second, the possibility that tumor cells might arise from normal stem cells; and third, the notion that tumors might contain “cancer stem cells” – rare cells with indefinite proliferative potential that drive the formation and growth of tumors [1].

The cancer stem cell shares many properties of the normal stem cell. It is generally accepted that normal stem cells show properties that provide for a long lifespan such as relative quiescence, resistance to drugs and toxins, and resistance to apoptosis. It ensues that cancer stem cells could also acquire these resistance mechanisms [8].

Basic cancer research has focused on identifying the genetic changes that lead to cancer. This has led to major advances in our understanding of the molecular and biochemical pathways that are involved in tumorigenesis and malignant transformation [1].

A tumor can be viewed as an aberrant organ initiated by a tumorigenic cancer cell that acquired the capacity for indefinite proliferation through accumulated mutations. If a tumor is considered particularly as an abnormal organ, then the principles of normal stem cell biology can be applied to understand better how tumors develop [1,9]. In agreement, previous work suggest that similarities between normal stem cells and tumorigenic cells may occur: a) Both normal stem cells and tumorigenic cells have extensive proliferative potential and the ability to give rise to new tissues; b) Both tumors and normal tissues are composed of heterogeneous combinations of cells, with different phenotypic characteristics and different proliferative potentials; c) Both normal stem cells and tumorigenic cells give rise to phenotipically heterogeneous cells that exhibit various degrees of differentiation [1]. In fact, tumorigenic cells can behave like stem cells that undergo an aberrant and poorly regulated process of organogenesis analogous to what normal stem cells do [10].

### The implications of cancer stem cells

Assuming that the growth of cancers is originated by cancer stem cells, it would have paramount implications for cancer therapy. Thus, the target of therapy must be to identify and kill this cancer stem cell population. If cancer stem cells can be identified and isolated, then we should be able to develop more efficiently new diagnostic markers and therapeutic targets expressed by the stem cells [1].

On the assumption that tumor growth and metastasis are driven by a small population of cancer stem cells, this might explain the failure to develop therapies that are consistently able to eradicate solid tumors. Despite the fact that presently available drugs can weaken metastatic tumors, these effects are usually short-term and often do not substantially extend the life of patients. One reason for the failure of these treatments is the acquisition of drug resistance by the cancer cells as they evolve; another possibility is that existing therapies fail to kill cancer stem cells effectively [1,11]. Existing therapies have been developed largely against the bulk population of tumor cells because they are often identified by their ability to shrink tumors. Even therapies that cause complete regression of tumors might spare enough cancer stem cells to allow regrowth of the tumors. [1]

### Drug resistance in cancer stem cells

It must be borne in mind that cancer stem cells are more resistant to chemotherapeutics than normal tumor cells. One particularly intriguing property of stem cells is that they express high levels of specific drug transporters. These transporters actively efflux drugs from cells, serving to protect them from cytotoxic agents [3]. Cancer cells can acquire resistance to chemotherapy by a range of mechanisms, including the mutation or overexpression of the drug target, inactivation of the drug, or elimination of the drug from the cell. Based on the tumor stem cell concept, an alternative model postulates that the cancer stem cells are naturally resistant to chemotherapy through their quiescence, their capacity for DNA repair, and drug transporter expression. As a result, at least some of the tumor stem cells can survive chemotherapy and support regrowth of the tumor [12]. Therapies that are more specifically directed against cancer stem cells might result in much more durable responses and even cures of metastatic tumors. Gene-expression profiling of cancer stem cells would permit the identification of the cells that are actually driving tumorigenesis. Isolating the cancer stem cells from the whole tumor that retain unlimited proliferative potential and to perform gene-expression profiling on those cells would be a more efficient way of identifying new therapeutic and diagnostic targets [1].

### Proof of concept

Cancer stem cells were found to be a population of cells with the ability to metastasize and form tumors. Cancer cells, must undergo self-renewal to become malignant. When stem cells divide, the division can give rise to a new stem cell as well as differentiated cells of the organ or tumor. Normally, the number of normal stem cells that are present in an organ are tightly regulated, but cancer stem cells lose this regulation, giving them the ability to self-renew and constantly expand themselves. Many oncogenic mutations target pathways that regulate proliferation and self-renewal [8,13,14].

Because stem cells can repair their DNA as they self-renew, they have the potential to accumulate mutations acquired after exposure to carcinogens. If tumors arise from stem cells, the accumulation of these mutations might be what we have come to recognize as the “multistep process of carcinogenesis” [15]. The cancer stem cell is related to but not identical to the normal counterpart. It is predicted that similar differences will be found in cell markers that will differentiate solid tumor stem cells from normal stem cells. We need to understand what the normal stem cell of a tissue looks like, and then we are in a position to compare the equivalent cancer stem cell to determine the genetic changes that occurred [8]. In fact, our group have shown that the cell-surface molecule AC133, a five transmembrane spanning 120 kD glycoprotein could be used a marker of progenitor cells in breast cancer. Furthermore, the expression of these EPC’s markers in a panel of breast tumors but not in the respective adjacent normal tissue highlights the importance of these cells as targets for breast cancer therapy. It is expected that these cells could be used as biomarkers of early diagnosis and treatment of breast cancer [16].

New lines of research include the identification of new markers that allow for the easy detection of the cancer stem cells, and the identification of new therapeutic targets that can be exploited to eliminate cancer stem cells from patients. Often therapy in the past has focused on proliferating cells, but in many cases the cancer stem cell will not be proliferating extensively. The ability to isolate the cancer stem cells will allow for the identification and testing of new therapeutic targets.

## Conclusions

There are many connections between stem cells and cancer that are important to understand. Studies of stem cells biology are lending insight into the origins of cancer and will ultimately yield new approaches to fight this disease.

The ideas forwarded in this review can be synthesized as following: a) self-renewal is the hallmark property of stem cells in normal and cancer tissues; b) cells that continue to divide over long periods of time are much more likely to accumulate mutations that cause cancer; c) in normal tissues that contain self-renewing stem cells the genetic changes also occur in the stem cells, or in progeny that acquire the potential for self-renewal.

Within most tumors there may exist cancer stem cells that can self-renew indefinitely, in contrast to most tumor cells that may have limited proliferative potential. Characterizing, identifying and isolating cancer stem cells could be a major step in the cure of cancer.
